# Identification of a New Serine Alkaline Peptidase from the Moderately Halophilic *Virgibacillus natechei* sp. nov., Strain FarD^T^ and its Application as Bioadditive for Peptide Synthesis and Laundry Detergent Formulations

**DOI:** 10.1155/2019/6470897

**Published:** 2019-11-30

**Authors:** Sondes Mechri, Khelifa Bouacem, Meriam Amziane, Ahlem Dab, Farida Nateche, Bassem Jaouadi

**Affiliations:** ^1^Laboratory of Microbial Biotechnology and Engineering Enzymes (LMBEE), Centre of Biotechnology of Sfax (CBS), University of Sfax, Road of Sidi Mansour Km 6, P.O. Box 1177, Sfax 3018, Tunisia; ^2^Laboratory of Cellular and Molecular Biology (LCMB), Microbiology Team, Faculty of Biological Sciences, University of Sciences and Technology of Houari Boumediene (USTHB), P.O. Box 32, El Alia, Bab Ezzouar, 16111 Algiers, Algeria; ^3^Biotech ECOZYM Start-up, Business Incubator, Centre of Biotechnology of Sfax (CBS), University of Sfax, Road of Sidi Mansour Km 6, P.O. Box 1177, Sfax 3018, Tunisia

## Abstract

A new peptidase designated as SAPV produced from a moderately halophilic *Virgibacillus natechei* sp. nov., strain FarD^T^ was investigated by purification to homogeneity followed by biochemical and molecular characterization purposes. Through optimization, it was determined that the optimum peptidase activity was 16,000 U/mL. It was achieved after 36 h incubation at 35°C in the optimized enzyme liquid medium (ELM) at pH 7.4 that contains only white shrimp shell by-product (60 g/L) as sole energy and carbon sources. The SAPV enzyme is a monomer protein with a molecular mass of 31 kDa as estimated by sodium dodecyl sulfate polyacrylamide gel electrophoresis (SDS-PAGE) and high-performance liquid chromatography (HPLC) gel filtration chromatography. The sequence of its NH_2_-terminal amino-acid residues showed homology with those of *Bacillus* peptidases S8/S53 superfamily. The SAPV showed optimal activity at pH 9 and 60°C. Irreversible inhibition of enzyme activity by diiodopropyl fluorophosphates (DFP) and phenylmethanesulfonyl fluoride (PMSF) confirmed its belonging to the serine peptidases. Considering its interesting biochemical characterization, the *sapV* gene was cloned, sequenced, and heterologously overexpressed in the extracellular fraction of *E. coli* BL21(DE3)pLysS. The biochemical properties of the recombinant peptidase (rSAPV) were similar to those of the native one. The highest sequence identity value (97.66%) of SAPV was obtained with peptidase S8 from *Virgibacillus massiliensis* DSM 28587, with 9 amino-acid residues of difference. Interestingly, rSAPV showed an outstanding and high resistance to several organic solvents than SPVP from *Aeribacillus pallidus* VP3 and Thermolysin type X. Furthermore, rSAPV exhibited an excellent detergent stability and compatibility than Alcalase 2.4 L FG and Bioprotease N100L. Considering all these remarkable properties, rSAPV has attracted the interest of industrialists.

## 1. Introduction

Despite advances in understanding the diversity and systematics of bacilli, studying their hydrolytic enzymes with bioengineering interest and their characterization has received more attention. Of particular interest, *Virgibacillus* is a genus of Gram-positive bacteria belonging to the wider family of *Bacillaceae* within the *Firmicutes* phylum [1]. The genus was named by Heyndrickx et al. [2]. At the time of writing, the genus *Virgibacillus* comprises 35 species with validly published names (http://www.bacterio.net/virgibacillus.html). Most members of genus *Virgibacillus* are mostly isolated from saline environments like marine sediment, soil, fish sauce fermentation, and lake [3–6]. The genus showed the ability to produce a great variety of extracellular hydrolytic enzymes. For instance*, Virgibacillus* sp. strain SK37, *Virgibacillus halodenitrificans* strain RSK, *Virgibacillus* sp. strain CD6, and *Virgibacillus dokdonensis* strain VITP14 have been shown to produce extracellular proteases [7–10]. However, information regarding stability and compatibility with laundry detergents and molecular modeling and structural characteristics, as well as the docking study of proteases from *Virgibacillus* is still very limited.

Peptidases or proteinases are known as enzymes able to cleave the array of proteins ingested into smaller peptide fragments in aqueous environments, but some peptidases perform slightly the peptide synthesis bonds in microaqueous media [11]. According to the Enzyme Commission (EC), peptidases belong to group 3 of the hydrolases and subgroup 4, hydrolysis of peptide bonds, but can still be classified according to the catalytic action (*endo*- or *exo*peptidase), active site, charge, molecular size, and substrate specificity.

In the last 50 years, proteases and other enzymes in laundry detergents switched from being minor additives to key ingredients [12, 13]. In common with all enzymes, external factors such as type of media, pH, and temperature are important for the catalytic efficiency, activity, stability, and proper functioning of proteases [14]. Therefore, the search for novel alkaline-stable and thermostable peptidases from extremophilic microorganisms and the engineering of the already available enzymes have been the major areas of research in this field over the years [15, 16]. Valuable characteristics of peptidases from polyextremophiles and their applications are gathered from a wide literature survey [17–19]. Salt-stable peptidases are the most widely used biocatalysts in laundry and dishwashing detergents where the high salt concentration is the desirable assurance of protease used in detergent, as NaCl is a key ingredient in the granulation process prior to addition of the protease in the detergent formulation [20, 21]. Research is in progress to isolate halophilic microorganisms that produce enzymes with this desired property [22, 23]. The assessment of bacterial diversity of an Algerian saline lake revealed the presence of a novel specie of a genus *Virgibacillus*, namely *Virgibacillus natechei* sp. nov., strain FarD^T^ with unusual phenotypic and genotypic characteristics [5]. In fact, the strain FarD^T^ was mesophilic, moderately halophilic, and alkaliphilic. This strain grew in the presence of NaCl concentrations ranging from 1 to 200 g/L, with an optimum at 100 g/L. The temperature range for growth was (15–40°C), with optimal growth occurring at 35°C. The pH range for growth was from 6 to 12, with an optimum at 7 [5]. No enzymatic research regarding this new species has been found in the literature, and for the first time with the current study, a research into the purification, characterization and biotechnological applicability of a new peptidase enzyme from strain FarD^T^ was investigated.

Herein, the current research was undertaken to purify, characterize, and to express for the first time, a new peptidase secreted from the culture supernatant of the moderately halophilic bacterium *Virgibacillus natechei* strain FarD^T^ and explore its promising potential enzymatic performance as a bioadditive for peptide synthesis biocatalysis and laundry detergent composition.

## 2. Materials and Methods

### 2.1. Materials

The raw material of shrimp shell was obtained in fresh conditions from a fishery market located at Sfax, Tunisia. Column chromatography materials were purchased from Agilent Technologies, Lawrence, Kansas, MO, USA. A Amersham LMW protein marker was purchased from GE Healthcare Europe GmbH, Freiburg, Germany. DNA molecular markers for electrophoresis and substrates were purchased from Invitrogen, Carlsbad (CA, USA) and Sigma Chemicals Co. St. Louis (MO, USA), respectively. All the microbiological media components were a product of Bio-Rad Laboratories (Hercules, CA, USA). Other chemicals and reagents used were of analytical grade. The used comparative enzymes were Thermolysin type X (Sigma-Aldrich Inc. Fluka, Chemical Co. St. Louis, MO, USA), Alcalase 2.4 L FG (Novozymes Biopharma DK A/S, Bagsvaerd, Denmark), Bioprotease N100L (Kerry Bioscience Ltd, Ireland, UK), and SPVP from *Aeribacillus pallidus* strain VP3 [17].

### 2.2. Methods

#### 2.2.1. Isolation, Identification, Phylogenetic Analysis, and Cultivation of Peptidase-Producing Strain FarD^T^

According to the phenotypic, morphologic, and molecular analysis, strain FarD^T^ is considered to represent a novel species of the genus *Virgibacillus* in the family *Bacillaceae* and order *Bacillales*, for which the name *Virgibacillus natechei* sp. nov., is proposed. The type strain of *Virgibacillus natechei* is FarD^T^ (DSM 25609^T^ or CCUG 62224^T^) [5]. As reported previously by the authors [5], strain FarD^T^ was maintained at 35°C on Sehgal and Gibbons medium (SG) containing the following (in g/L): casamino acids, 7.5; yeast extract, 10; sodium glutamate, 1; trisodium citrate, 3; MgSO_4_·7 H_2_O, 20; KCl, 2; FeSO_4_·7 H_2_O, 0.036; and MnCl_2_·4 H_2_O, 0.00036 supplemented with 100 g/L of NaCl at pH 7. After incubation at 35°C, a halo of casein degradation was revealed around the colony growth onto skimmed milk agar plates, as well described previously by the authors [17, 18]. The protease-producing isolate was propagated on SG agar plates at 35°C and conserved at −80°C in SG medium where 20% of glycerol was added.

#### 2.2.2. Peptidase Production

The preculture of strain FarD^T^ was carried out in a 1 L Erlenmeyer flask containing 100 mL of SG liquid medium at pH 7 supplemented with 100 g/L NaCl and incubated at 35°C overnight. This preculture was used to inoculate the culture using the optimized ELM media at pH 7.4 containing only 60 g/L white shrimp shell by-product in the Erlenmeyer flask. The initial A_600_ nm of the culture was 0.1. The flasks were incubated at 35°C for 36 h with shaking at 200 rpm in 1000 mL Erlenmeyer flasks with a working volume of 100 mL. Bacterial growth of strain FarD^T^ and cell dry weight were estimated as detailed recently [24]. The cell pellet was removed before each assay, and the obtained clear supernatant was served as a peptidase source in the subsequent investigations.

#### 2.2.3. Peptidase Activity Assay

The peptidase activity was assayed by the addition of 0.5 mL of an appropriately diluted enzyme in 0.1 M glycine-NaOH buffer at pH 9, supplemented with 2 mM CaCl_2_ (buffer A), to a 0.5 mL of 10 g/L casein. The reaction mixture was incubated for 15 min at 60°C. The reaction was stopped by adding 0.5 mL of 200 g/L TCA. In fact, the liberated tyrosine from casein was measured using the Kembhavi method [25]. One unit (U) of peptidase was defined as the amount of enzyme releasing 1 *µ*g of tyrosine released under the assay conditions detailed.

Peptidase activity present in the laundry detergent solution was determined through the method proposed by Boulkour Touioui et al. [26] which used the *N*,*N*-dimethylated casein (DMC) as a substrate and 2,4,6-trinitrobenzene sulfonic acid (TNBSA) as a colour indicator. One unit of protease activity was defined as the amount of enzyme required to catalyze the cleavage of 1 *µ*mole of peptide bond from DMC per minute under the experimental conditions used. The absorbance was measured at 450 nm.

#### 2.2.4. Peptidase Purification Procedure

500 mL of a 36 h culture of strain FarD^T^ was centrifuged for 20 min at 10,000 ×g. The clear supernatant containing extracellular peptidase was used as the crude enzyme preparation and was submitted to the following purification steps. Proteins were precipitated to 20% with solid (NH_4_)_2_SO_4_ and then centrifuged at 10,000 ×g for 20 min. The obtained supernatant was saturated up to 80% with (NH_4_)_2_SO_4_ and centrifuged, the precipitate was resuspended in a minimal volume of buffer B composed with 50 mM MOPS and 2 mM CaCl_2_ at pH 7, and dialyzed overnight. Next, the obtained supernatant was loaded and applied to an HPLC system using a ZORBAX PSM 300 HPSEC column previously equilibrated with buffer B. Pooled fractions, containing peptidase activity, were concentrated for further analysis.

#### 2.2.5. Analytical Methods

Protein concentration was estimated through the method of Bradford [27], using bovine serum albumin as a reference. The molecular weight of the purified peptidase SAPV was carried out by SDS-PAGE, as described by Laemmli [28]. Casein zymography staining was estimated by incorporating azo-casein (10 g/L) into the separating gel before polymerization, as detailed elsewhere [29]. Bands of purified SAPV were separated on SDS gels, transferred to a ProBlott membrane, and then the NH_2_-terminal sequence analysis was performed by automated Edman degradation using an Applied Bio system Model 473A gas-phase sequencer.

#### 2.2.6. Effects of Inhibitors, Reducing Agents, and Metal Ions on SAPV Stability

Preincubation of the purified SAPV with specific inhibitors, reducing agents, and various and divalent metal ions was investigated and described by Jaouadi et al. as well [29]. The remaining peptidase activity was measured after preincubating the purified SAPV with each compound for 1 h at 40°C. The remaining SAPV activity was measured by optimum assay. For the effect of metal ions, the nontreated SAPV, or dialyzed, was considered as control 100%. For the inhibitors and reducing agents on SAPV stability, the peptidase solution without inhibitor was considered as control.

#### 2.2.7. Determination of the Effects of pH and Temperature on SAPV Activity and Stability

Determination of the optimum pH of SAPV activity was performed at 60°C with different buffer systems at 0.1 M each supplemented with 2 mM calcium. The pH stability of SAPV peptidase was determined by preincubating the enzyme in buffer solutions with different pH values for 210 min at 40°C. The following buffers were used: glycine-HCl (pH 3–5), 2-(*N*-morpholino)ethanesulfonic acid (MES) (pH 5-6), piperazine-*N*,*N*′-bis(2-ethanesulfonic acid) (pH 6-7), 4-(2-hydroxyethyl)-1-piperazineethanesulfonic acid (HEPES) (pH 7-8), Tris-HCl (pH 8-9), glycine-NaOH (pH 9–11), bicarbonate-NaOH (pH 11–11.5), sodium phosphate dibasic-NaOH (pH 11.5–12), and potassium chloride-NaOH (pH 12-13).

Using casein as a substrate, the effect of temperature on SAPV was investigated at 30–80°C at pH 9. Its thermostability was examined by incubation for 600 min at 40–70°C at pH 9 with and without 2 mM CaCl_2_. Aliquots were withdrawn at desired time intervals to test the remaining activity. The nonheated SAPV was considered as a control. Samples were taken out sequentially at various time intervals and residual activity was analyzed by performing the assay under standard assays.

#### 2.2.8. Determination of the Effect of Polyols on SAPV Stability

The effect of variety of polyols, with concentration of 100 g/L, on SAPV thermostability was carried out by its incubation at 80°C for 30 min with and without different polyols. The combination of glycerol (100 g/L) and calcium (2 mM) on the thermostability of SAPV peptidase was monitored at 80°C for 450 min. Peptidase assays were carried out under the standard experimental assay conditions.

#### 2.2.9. Effect of NaCl and KCl on SAPV Activity

SAPV was preincubated for 1 h with various concentrations of NaCl and KCl from 0.5 to 5 M at pH 9. The activity of SAPV without NaCl or KCl was taken as 100%. The residual peptidase activity was measured by performing the assay under standard conditions.

#### 2.2.10. Molecular Study of SAPV Protease

*(1) Cloning and Expression of the SapV Gene*. Based on the high homology found in peptidase S8 gene from *Virgibacillus massiliensis* DSM 28587, two primers (F-MS40 and R-MS41) were designed to generate ∼1.4 kb PCR (Polymerase Chain Reaction) fragment encoding the peptidase SAPV. The PCR was carried out in a MultiGene™ OptiMax Thermal Cycler, 96 × 0.2 mL (Diamed Lab Supplies Inc., Canada), and the mixture (100 *µ*L) was composed of 250 ng of DNA template, amplification buffer, 30 pg of each primer, and 2 U of *pfu* (Biotools, Madrid, Spain). The PCR cycling parameters were 94°C for 5 min, followed by 35 cycles of 94°C for 60 s, 58°C for 60 s, and 72°C for 45 s as well as 72°C for 10 min. The PCR product was purified and cloned into pCR-Blunt, pUT57, and pTrc99A Vectors, previously digested with *Eco*RI, leading to the pSM40, pSM41, and pSM42 plasmids, respectively, using the *E. coli* BL21(DE3)pLysS [29].

For the recombinant SAPV production, the *E. coli* BL21(DE3)pLysS/pSM41 and *E. coli* BL21(DE3)pLysS/pSM42 were induced by the addition of 5 mM IPTG after reaching an absorbance (A_600_) of about 0.7. The enzyme crude extracts were obtained from the extracellular fraction by the modified procedure described by Abe et al. [30]. The extracellular recombinant enzyme (rSAPV) was purified to homogeneity using the same steps applied for the purification of the native enzyme.

*(2) Sequence Analyses and Bioinformatics Tools*. The nucleotide sequence of *sapV* gene was determined at least with three positive clones on both strands, using the automated DNA sequencer ABI PRISM® 3100-Avant Genetic Analyser (Applied Biosystems, Foster City, CA, USA). Multiple and alignments analyses of nucleotide and protein sequences were performed using the available program tools at the ExPASy database (https://www.expasy.org/).

#### 2.2.11. Enzymatic Performance of the Purified Peptidase rSAPV

*(1) Substrate Specificity Determination*. The substrate profile specificity of rSAPV from *E*. *coli* BL21(DE3)pLysS/pSM42 was determined using natural and modified protein substrates, as well as esters and synthetic peptides. Enzymatic activities were determined on each substrate based on standard conditions, previously described elsewhere [31, 32].

*(2) Kinetic Characterization of Purified and Commercial Proteases*. Kinetic parameter constants, *K*_*m*_ and *V*_max_, were determined by Lineweaver–Burk plots using casein and Suc-F-A-A-F-*p*NA as substrates at different concentrations ranging from 0.10 to 50 mM for 15 min at optimum pH and temperature of each commercial or purified peptidases (rSAPV, Thermolysin type X, Alcalase 2.4 L FG, and Bioprotease N100L) at a final concentration of proteins 1.5 mg/mL.

*(3) Effects of Organic Solvents on rSAPV, SPVP, and Thermolysin Type X Peptidases*. Various organic solvents, with different log *P* values at 50% (v/v), were tested as well detailed in a previously study [17]. The peptidase enzymes used were rSAPV, Thermolysin type X, and SPVP. Enzyme assays were carried out under the standard assay conditions for each used peptidase (60°C and pH 9 for rSAPV, 70°C and pH 8 for Thermolysin type X, and 60°C and pH 10 for SPVP).

*(4) Comparison of Stability and Compatibility with Laundry Detergents between rSAPV and Commercial Proteases*. Using DMC as a substrate, the compatibility of rSAPV, Alcalase 2.4 L FG, and Bioprotease N100L enzymes with a wide range of commercialized detergents was assayed. So, to simulate washing conditions, detergent solutions were prepared in tap water (as laundry washing has to be done in tap water) at a concentration of 7 g/L and then heated for 1 h at 70°C to destroy the indigenous protease or enzyme activity anterior to the addition of the enzymes. Then, residual peptidase activity was analyzed by performing the assay under standard protocol. The peptidase activity of a control (without any detergent), incubated under similar conditions, was taken as 100%.

*(5) The Effectiveness of Protein Stain Removal from Cotton Fabrics*. To estimate the stain removal capabilities of rSAPV, Alcalase 2.4 L FG, and Bioprotease N100L proteases, clean white cotton cloth pieces (5 cm × 5 cm) were soaked and dried with chocolate, egg, and blood. The used blood was freshly obtained from a local municipal slaughterhouse and collected from cow into anticoagulant heparin tubes (Sfax municipal slaughterhouse, permission was obtained from this slaughterhouse to use these animal parts). The stained cloth pieces were shake-incubated separately for 1 h with litre beakers containing a total volume of 100 mL of tap water, Class detergent (7 g/L, in tap water), and detergent added with 500 U/mL of rSAPV peptidase or with 500 U/mL of commercial proteases (Bioprotease N100L and Alcalase 2.4 L FG), followed by rinsing with water. Then, the washed cloth pieces were dried. Visual examination of various pieces was also carried out to show the effect of each used peptidase in the removal of proteinaceous stains. The untreated stained piece of cloth was taken as a control.

## 3. Results and Discussion

### 3.1. Producing Strain FarD^T^ Peptidase Using Powder from White Shrimp Shell By-Product

**Figure 1:**
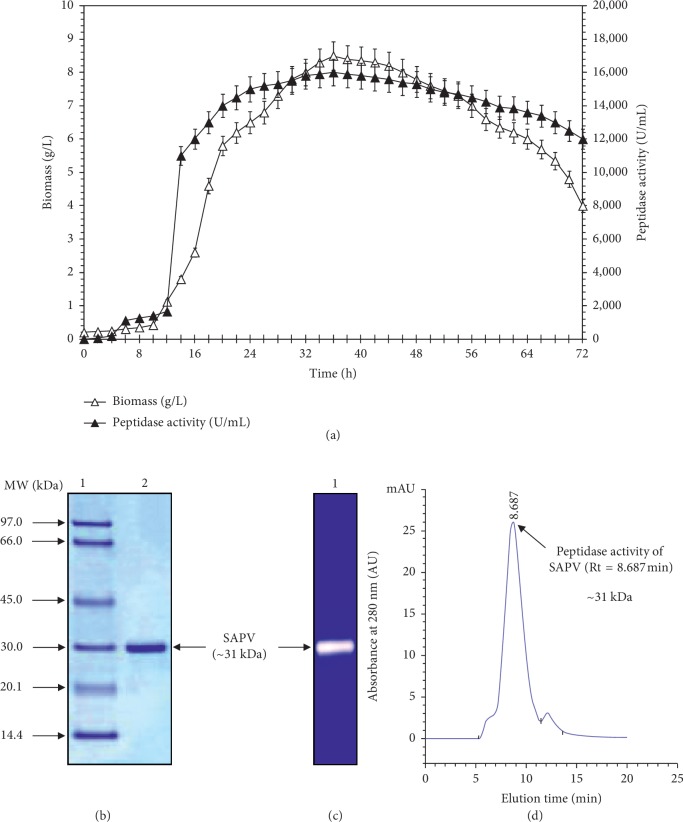
(a) Time course of *Virgibacillus natechei* strain FarD^T^ cell growth (Δ) and peptidase production (▲). Cell growth was monitored by measuring the absorbance at 600 nm and was converted to cell dry weight (g/L). Each point represents the mean of three independent experiments. (b) 12% SDS-PAGE of the purified peptidase SAPV. Lane 1, purified SAPV (50 *µ*g) obtained after ZORBAX PSM 300 HPLC chromatography (Rt = 8.676 min). Lane 2, Amersham LMW protein marker. (c, d) Zymogram caseinolytic activity staining of peptidase activity. Lane 1, the purified peptidase SAPV (50 *µ*g).

Strain FarD^T^ was cultured for 36 h at 35°C in Erlenmeyer flasks in optimized ELM media. A high level of peptidase production (16,000 U/mL) was obtained with this bio waste as the sole carbon and nitrogen source (Figure 1(a)). Thus, the powder from white shrimp shell by-product is a source for the development of the bacterium and the synthesis of metabolites as well. While *Virgibacillus pantothenticus* strain MTCC 6729 was demonstrated to produce only 18.2 U/mL using casein at 40°C after 72 h of incubation at pH 9 [33], *Virgibacillus dokdonensis* strain VIT P14 was demonstrated to produce 996 U/mL using zobell marine broth [34]. To an increasing extent, by-products from shrimp shell by-product are considered by researchers as a “raw material” and not a “waste material.” Yet, the biochemists and microbiologists used it as a cheap substrate for the production of bioactive substances such as enzymes and peptides [35–37]. Annamalai recorded the production of halostable peptidase enzyme from halophilic bacteria using marine bio-wastes such as squid pen powder and crab and shrimp shells [38, 39].

### 3.2. SAPV Purification

The summary of the peptidase SAPV purification is tabulated in Table 1. The recovery yield and purification fold of SAPV from strain FarD^T^ are higher than those of peptidases from *Aeribacillus pallidus* strain VP3 and from *Virgibacillus dokdonensis* strain VIT P14 [17, 34]. In fact, the protease from strain VIT P14 was purified with a purification fold of 5.3 and by a low recovery yield of about 9, while the SPVP was purified by a recovery yield and purification fold, respectively, of about 22% and 18. The specific activity displayed by SAPV (74,652 U/mg) was significantly high compared with that reported from strain VP3 (41,250 U/mg) and strain VIT P14 (102 U/mg). Based on these results, SAPV enzyme seems very promising and can be tested in several biotechnological applications.

### 3.3. Molecular Weight Determination

The SDS-PAGE analysis of the pooled fractions from the HPLC column showed one band, with apparent molecular mass, about 31 kDa (Figure 1(b)). Similarly, Rajeswari et al. reported that the purified protease from strain VIT P14 had a molecular weight of 36 kDa [34]. The proteases from extremophilic bacteria showed a molecular weight ranged from 40 to 130 kDa [40]. Zymogram activity staining revealed the presence of one prominent zone of caseinase activity for the purified peptidase (Figure 1(c)). The SAPV preparation was a homogeneous pure enzyme with high purity confirmed by a symmetrical peak with Rt of 8.687 min (Figure 1(d)), matching with the protein of ∼31 kDa (Figure 1(b)). The results mentioned above strongly suggested that SAPV is a monomeric enzyme comparable to previous investigation for peptidases from strains VP3 [17] and VIT P14 [34].

### 3.4. NH_2_-Terminal Amino Acid Sequence Analysis of SAPV

The blotted peptidase SAPV was submitted to NH_2_-terminal sequencing, which allowed the identification of 25 residues, which are EQTVPWGIDYIGKAAAHQLGIFGKG. The sequence showed uniformity, indicating that SAPV was isolated in the pure form. The NH_2_-terminal sequence of SAPV was compared with other closely related peptidase sequences and noted to share greatest homology with those from *Bacillus* spp. and related genus. So, the amino acid resemblance of the NH_2_-terminal sequence between these proteases is an indication of the existence of a molecular relationship and that all these enzymes seem to derive from the expression of an ancestral gene. As illustrated in Table 2, the identity reached 80% with peptidase S8 from *Virgibacillus massiliensis* DSM 28587, which contains five modified residues at positions T3L, G7N, K13S, A14T, and F22S, respectively. These results and comparison with the nearest proteases indicated that SAPV enzyme from *Virgibacillus natechei* strain FarD^T^ is a new peptidase.

### 3.5. Biochemical Characterization of SAPV Peptidase

#### 3.5.1. Effects of pH on SAPV Activity and Stability

**Figure 2:**
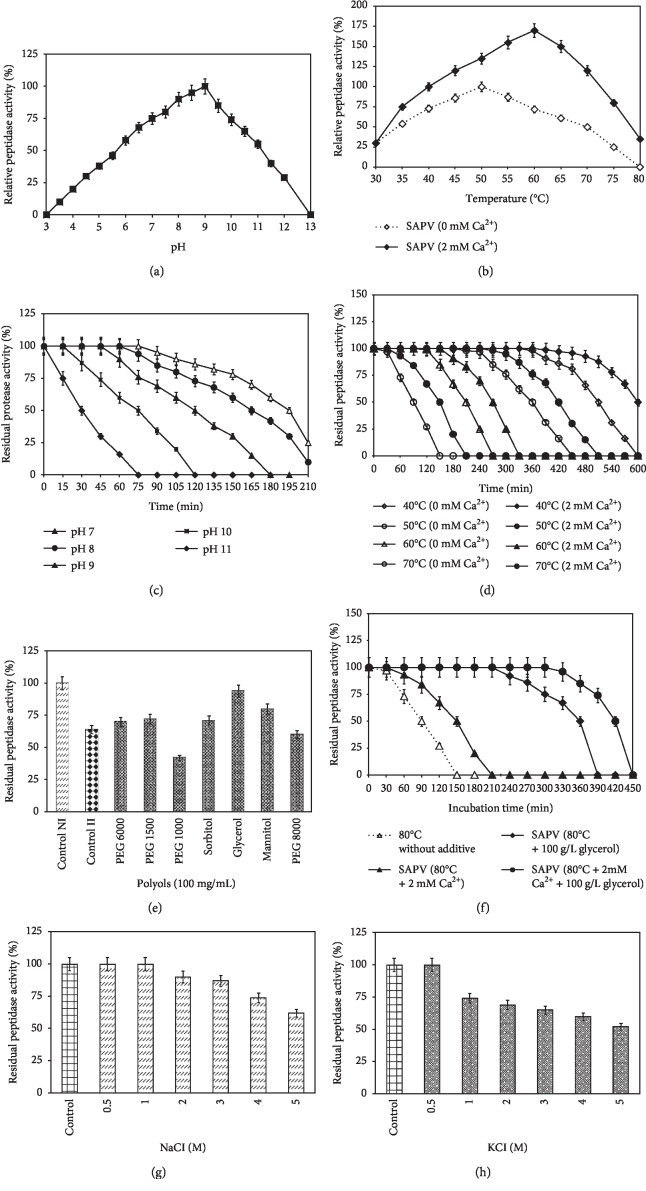
Physicochemical properties of the purified SAPV from strain FarD^T^. Effects of pH (a) and the temperature (b) on the activity of SAPV. Effects of the pH stability (c) and thermostability (d) of SAPV. The enzyme was preincubated in the presence and absence of CaCl_2_ at 40, 50, 60, and 70°C. The activity of the nonheated peptidase was taken as 100%. Each point represents the mean of three independent experiments. (e) Stability of SAPV in the presence of various polyols at 100 mg/mL. Peptidase activity of the control sample, without additive, incubated under similar conditions, was taken as 100%. Vertical bars indicate standard error of the mean (*n* = 3). (f) Effect of the thermostability of SAPV at 80°C. The peptidase was preincubated in the absence (▲) or presence of additive: 2 mM Ca^2+^ (♦); 100 g/L glycerol (⯀); and 2 mM Ca^2+^ and 100 g/L glycerol (●). The residual protease activity was determined from 0 to 450 min at 30 min intervals. The activity of the nonheated peptidase was considered as 100%. Each point represents the mean (*n* = 3) ± standard deviation. Effect of NaCl (g) and KCl (h) from 0.5 to 5 M on the stability of the purified SAPV.

According to the results presented in Figure 2(a), SAPV possesses an optimum activity at pH 9. The relative activity at pH 8 and 10 were, respectively, 90 and 74%. At pH levels of 5 and 6, the SAPV activity was 38 and 58%, respectively, while the two proteases produced by *Virgibacillus dokdonensis* strain VITP14 and *Virgibacillus pantothenticus* strain MTCC 6729 showed, respectively, an optimal pH at 10 and 7 [10, 33, 34]. The protease activity secreted by strain MTCC 6729 lost 20% of its activity at pH 12 and was reduced to 27% at pH 6 [33]. These differences mentioned between the proteases of the bacteria produced by the same genus indicated that the range of pH activity is in relation with the source of the strain from which it has been isolated.

The effect of pH on the stability of SAPV was determined at 60°C. The pH stability profile of SAPV, illustrated in Figure 2(c), indicated that the peptidase showed a maximum activity at pH 7, 8, 9, and 10, respectively, until 75, 60, 45, and 15 min. The half-lives (*t*_1/2_) of SAPV are 195, 165, 120, 75, and 30 min, respectively, at pH of 7, 8, 9, 10, and 11. At pH values between 7 and 10, SPVP activity is 100% after 24 h of incubation [17]. In agreement to the current study, SAPRH protease, produced by *Bacillus safensis* strain RH12, which has an optimum pH at 9 and stable in the pH range of 7–12, has been applied in the laundry industry [24]. Rather, according to Gupta et al., a protease which is useful for detergent application should be active in a pH range between 8 to 12 [41]. So, SAPV can greatly be tested as a bioadditive in solid and liquid laundry detergents.

#### 3.5.2. Effects of Temperature on SAPV Activity and Stability

As shown in Figure 2(b), SAPV retained its activity over a wide range of temperature between 30 and 80°C with a maximum activity at 60°C. With 2 mM of Ca^2+^, SAPV activity was enhanced to 135 and 120%, respectively, at 50 and 70°C, with an optimum at 60°C. 35% of its activity was retained at 80°C. Without 2 mM of Ca^2+^, SAPV was observed to retain only 72 and 50% of activity, respectively, at 60 and 70°C, with an optimum at 50°C.

In agreement to the current study, SPVP has an optimum temperature at 60°C [17], the same of SAPV. Otherwise, the two peptidases produced by *Virgibacillus dokdonensis* strain VITP14 and *Virgibacillus pantothenticus* strain MTCC 6729 showed, respectively, an optimal temperature at 40 and 50°C [10, 33, 34]. The SAPRH protease successfully incorporated in commercial liquid laundry detergent (Class) of the STE JMAL (EJM)-Laundry Detergent Industry (Tunisia) showed the same optimum temperature of that of SAPV [24]. The calcium-dependent stability of SAPV was determined by incubating the peptidase at different temperatures from 40 to 70°C for 600 min in the presence and absence of 2 mM Ca^2+^ at pH 10 (Figure 2(d)). The *t*_1/2_ of SAPV in the absence of any additive was 510, 360, 210, and 90 min at 40, 50, 60, and 70°C, respectively. Adding calcium to 2 mM, the *t*_1/2_ of SAPV was enhanced to 600, 420, 270, and 150 min, respectively. This behavior was similar to what has already been reported for SPVP protease, when the *t*_1/2_ was ameliorated from 6 to 8 h when adding 2 mM of Ca^2+^ [17]. All the evoked results suggested the high thermostable nature of SAPV peptidase in alkaline conditions.

#### 3.5.3. Effects of Inhibitors and Metal Ions on Peptidase Stability of SAPV

The findings in Table 3 revealed that SAPV peptidase is completely inhibited by DFP and PMSF, well-known serine peptidase inhibitors. The strong inhibition by those two inhibitors suggested that a serine residue was involved in the active site of the peptidase SAPV. Likewise, the protease from strain VIT P14 showed a complete inhibition against PMSF after an incubation at 37°C for 30 min [34]; however, the protease from strain MTCC 6729 retained 35% of its activity against 5 mM PMSF and totally inhibited against 10 mM PMSF [33]. The essential serine residue was often described for the alkaline salt-stable proteases from the halophilic *Bacillus* strains [22, 23], while the peptidase SAPV retained all of its activity towards the trypsin competitive reagents, the chymotrypsin alkylating agent, the zinc protease inhibitor, and the acid and thiol reagents. In fact, the SBTI, TLCK, TPCK, benzamidine, DTNB, ld-DTT, 2-ME, NEM, iodoacetamide, leupeptin, pepstatin A, MIA, EPNP, and 1,10-phenanthroline monohydrate had almost no effect on the activity. This behavior is also detailed in SPVP protease [17]. The protease from strain MTCC 6729 is slightly inhibited by *β*-ME and ld-DTT, which are two thiol inhibitory proteases [33]. In addition, the peptidase from strain VIT P14 showed a moderate inhibition with ld-DTT. In fact, the relative activity is 89% in the presence of ld-DTT [34]. With the ethylene-diaminetetraacetic acid (EDTA) and ethylene glycol-bis (*β*-aminoethyl ether)-*N*,*N*,*N′*,*N′*-tetraacetic acid (EGTA), as chelating agent inhibitors, the SAPV peptidase retained 86 and 74% of its activity, respectively, in the presence of 10 mM EDTA and 2 mM EGTA, which suggests that no metal cofactors were required. Or, the two inhibitors are widely used to sequester metal ions. So, the moderate inhibition by EDTA and EGTA suggested that SAPV peptidase is not a metalloprotein.

These observations indicate that SAPV enzyme showed a potential for use as a detergent bioadditive. Indeed, the chelating agents function as water softeners in commercial detergents and assist in stain suppression [42]. In Table 3, the influence of several metal ions on SAPV peptidase is tabulated. The addition of Ca^2+^ increased SAPV activity by 290%. The Ca^2+^ has been shown as ions that speed up the peptidase activity. Similarly, the addition of 2 mM Ca^2+^ increased SPVP activity by 283% of the control [17]. Recently, with a docking study, two calcium binding sites have been shown at SAPRH protein, which play a significant role in stabilizing the enzymatic activity by binding at specific sites [24]. In the same context, SAPV peptidase is improved by Fe^2+^, Cu^2+^, and Zn^2+^ at 2 mM in comparison with the control by 210, 125, and 120%, respectively. However, the Fe^2+^ and Cu^2+^ have an inhibitory effect on the peptidase activity from train VIT P14 [34]. The heavy metals: Co^2+^, Hg^2+^, and Cd^2+^ completely inhibited SAPV activity. These observations were similar to that described to the SPVP protease which is totally inhibited by Co^2+^, Cd^2+^, and Hg^2+^ at 2 mM. This phenomenon is in contrary to what has been described in the peptidase produced by strain VIT P14, which, Co^2+^ showed a stimulatory effect on activity [34].

#### 3.5.4. Impact of Polyols on SAPV Activity and Thermal Stability

The results shown in Figure 2(e) mentioned that the highest level of SAPV peptidase was registered with glycerol followed by mannitol and PEG 1500 as additives, at a concentration of 100 g/L. Over and above, the thermal stability was more effective by the presence of calcium at 2 mM and xylitol at 100 g/L together. In fact, the half-life of SAPV at 80°C without any additive is 90 min; contrariwise, it is 420 min by the combined effect of calcium and glycerol (Figure 2(f)). The addition of polyols has already improved the thermal stability of SPVP. In fact, the half-life times at 80°C was determined to be, respectively, 9 h with calcium at 2 mM and xylitol compared with 2 h in the absence of any additive [17]. Researchers have assumed this phenomenon of enhanced stability of enzymes with polyols at high temperatures is related to their hydroxyl groups [24, 43].

#### 3.5.5. Osmolyte Effect of the SAPV Thermostability

The effect of some osmolytes on the SAPV thermostability was performed using NaCl and KCl at 0 to 5 M at 60°C for 1 h. Results showed that more than 50% of residual activity was obtained at the concentration of 0.5–5 M of both osmolytes (Figures 2(g) and 2(h)). At 5 M NaCl and KCl, peptidase activity decreased progressively and retained 62 and 52% of its residual activity, respectively. A similar kind of high-salt tolerance and osmotic pressure is a distinctive quality of halophiles proteases, which have a number of future purposes in industrial bioprocess [22, 23, 44].

### 3.6. Cloning and Heterologous Expression of the sapV Gene

#### 3.6.1. Cloning and Sequencing of the sapV Gene

**Figure 3:**
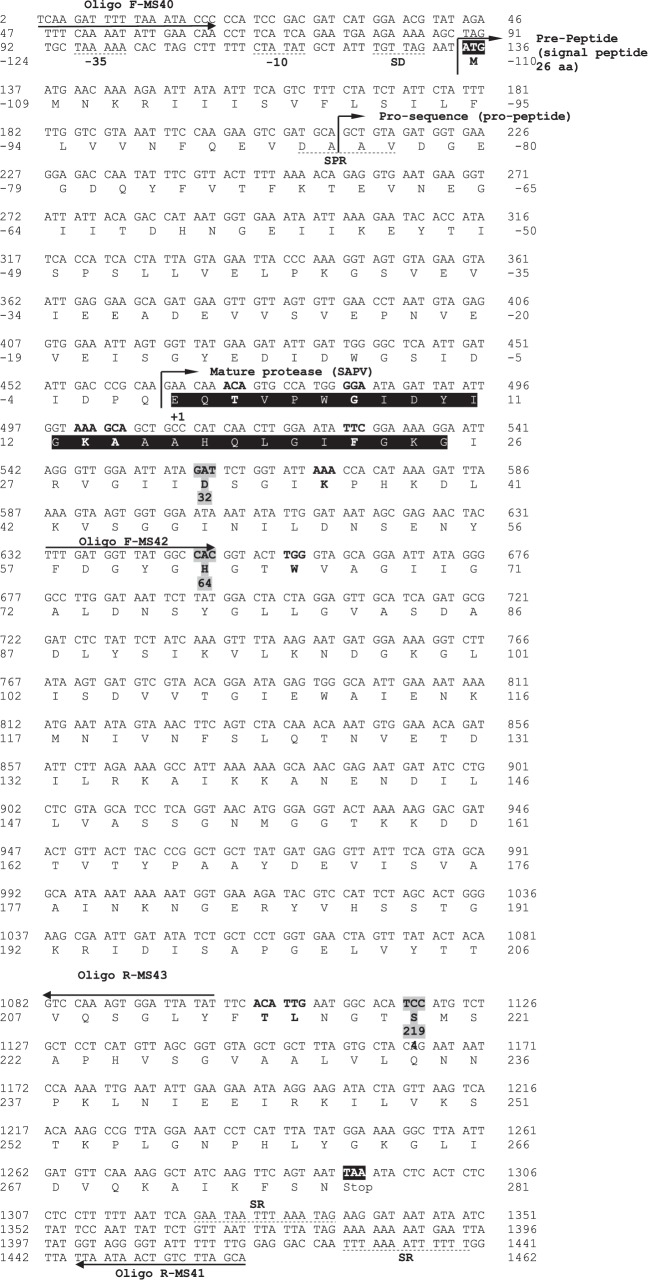
Nucleotide and deduced amino acid sequences of the *sapV* gene (GenBank accession number: **MN094794**). The *sapV* consisted of 1158-bp encoding a polypeptide of 386 amino acid residues. Translation starts at a nucleotide position 1. The first amino acid of the mature protease, Glu, is counted as +1. Numbers written on both sides of the lines indicate the positions of nucleotides and amino acids. The nucleotide sequences ATG and TAA (both highlighted) represent the initiation and terminal codon of translation, respectively. The positions of the four used foreword (F-MS40 and F-MS42) and reverse (R-MS41 and R-MS43) primers were underlined. The black box stands for the NH_2_-terminal amino acid sequence of the purified SAPV. SD: Shine–Dalgarno-like sequence. SPR: signal peptide recognition site. SR: stacking region.

The PCR product corresponding to the entire SAPV coding region was cloned and sequenced leading to the pSM40. It revealed an open reading frame (ORF) of 1158 bp encoding 386 amino acids (Figure 3). This ORF was checked as the gene encoding SAPV peptidase enzyme, as given by the Edman degradation mechanism, the deduced amino acid sequence was noted to comprise the 25 NH_2_-terminal amino acid sequence of the purified enzyme.

The signalP expected a signal peptide (pre-sequence) of 27 amino acids bounded with the signal peptidase recognition (SPR) site D-A-A-V, marking that a group of robustly hydrophobic amino acids was maintained. Belonging to the signal sequence, the prosequence consisting of 84 amino acids had to be brooked by autoproteolytic processing in the periplasm. The active mature SAPV peptidase consisted of 276 amino acids having a predicted molecular weight of 29,556.68 Da. The apparent molecular weight of the purified enzyme (∼31 kDa) determined by SDS-PAGE and HPLC gel filtration chromatography was in good agreement with the predicted value. The typical triad catalytic residues (D32, H64, and S219) in the active site and three serine peptidase signatures (amino acid residues 28–39, 64–75, and 216–226) [45] were also conserved in the *sapV* gene.

The amino acid sequence deduced from the nucleotide sequence of the *sapV* gene was compared with that of other known subtilisin superfamily of serine peptidases S8/S53. The amino acid composition of SAPV indicated that it was devoid of cysteine and cystine residues. The most significant feature of the amino acid composition of SAPV was its high Asx amount (Asp and Asn residues), compared with other subtilisins. SAPV contained 37 amino acid residues of Asx (17 Asp and 20 Asn), corresponding to 13.4 mol%, whereas SAPB [29], subtilisin Novo [46], and subtilisin Carlsberg [47, 48] possess exclusive 12, 10.2, and 9.45 mol%, respectively.

#### 3.6.2. Expression of the sapV Gene in *E. coli* and Characterization of Recombinant Enzyme rSAPV

To express SAPV, the corresponding gene was cloned downstream of P*T7* and P*tac* promoters in pSM41 and pSM42, respectively and then transformed into *E. coli* BL21(DE3)pLysS strain. No alkaline peptidase activity was found in the periplasmic fraction, neither in the intracellular fraction for all recombinant strains. Relatively elevated quantities of specific activity of 310 and 1,250 U/mg were, nevertheless, detected in the extracellular fractions of *E. coli* BL21(DE3)pLysS/pSM41 and *E. coli* BL21(DE3)pLysS/pSM42, respectively. Found on this investigation, the SAPV peptidase was most efficiently expressed with the construction of P*tac*-*sapV* (pSM42). The latest was, therefore, preserved for the purification of the recombinant peptidase (rSAPV). Extracellular rSAPV (pSM42) was purified using the same steps taken for the native enzyme from strain FarD^T^. All the biochemical properties from rSAPV were almost analogous to those of the original one. The recombinant enzyme, rSAPV, can be readily prepared on a large-scale for biotechnological sectors.

The protein sequence of SAPV showed 97.66, 64.06, 64.06, and 40.10% identity with peptidase S8 from *Virgibacillus massiliensis* [WP_051739338], *Aeribacillus pallidus* [WP_094244502], *Anoxybacillus flavithermus* [WP_088223339], and *Paenibacillus alvei* [WP_005550669], respectively. Furthermore, the highest sequence identity value (97.66%) of the SAPV was obtained with peptidase S8 from *Virgibacillus massiliensis* [WP_051739338], with 10 amino acids of difference. Even so, one amino acid in the signal peptide, and 9 amino acids (T3L, G7N, K13S, A14T, F22S, K36N, W65K, T214L, and L215A) in the mature SAPV were recorded to differ from the peptidase S8 from *Virgibacillus massiliensis*. In spite of displaying high levels of homology, this peptidase S8 proved relatively dissimilar properties. As a matter of fact, they indicated a sharply different physicochemical and kinetic properties as compared with SAPV. It can, therefore, be inferred that the substitutions in the mature SAPV, noted to differ from the other peptidases, synchronously enhanced the alkaline pH, thermostability, and catalytic parameters of the enzyme.

### 3.7. Functional Properties of the Purified rSAPV

#### 3.7.1. Substrate Specificity Profile

The ability of proteolytic enzyme to hydrolyze and act upon a specific class of substrates with flawless precision and efficiency is a fundamental feature of biotechnology application. The substrate specificity is frequently assigned to the amino acid residues foregoing the peptide bond they broke. The relative hydrolysis rates of various protein substrates were investigated to elucidate the amino acid preference/substrate specificity of rSAPV enzyme from *E*. *coli* BL21(DE3)pLysS/pSM42 (Table 4). When assayed with esters as substrates, rSAPV showed high level of peptidase activity towards ATEE, followed by BTEE, which are two N-terminal- and C-terminal-protected l-tyrosine. Contrariwise, no esterase activity detected with BAEE, BCEE, and TAME as substrates which are N-terminal and C-terminal protected l-arginine and cysteine. Thus, rSAPV exhibits both amidase and esterase activities and preferentially hydrolyze peptide bonds that are contributed by hydrophobic and cationic amino acids at *P*_1_. The specificities for synthetic substrates at N-terminal residues to the cleavage site (*P*_1_, *P*_2_, etc) were also investigated, see Table 4. rSAPV largely preferred Suc-F-A-A-F-*p*NA peptide with 100% of activity. The relative peptidase activities are 87, 75, 62, and 52% on Suc-A-A-P-F-*p*NA, Suc-A-A-V-A-*p*NA, Suc-A-A-P-M-*p*NA, and Suc-L-L-V-Y-*p*NA, respectively. So, rSAPV peptidase chiefly preferred hydrophobic substrates, especially those with aromatic amino acids occupying the *P*_1_ and *P*_4_ positions of *p*NA peptides. When alanine amino acid is in the *P*_1_ position, the relative activity of rSAPV is 38, 35, and 21 for Suc-A-A-P-L-*p*NA, Suc-A-A-F-*p*NA, and Suc-A-A-V-*p*NA, respectively. Thus, rSAPV was closely dependent in terms of specificity for position *P*_1_, but also with regard to the effects of amino acid residues neighboring the cleavage site.

#### 3.7.2. Determination of Kinetic Parameters

The kinetic parameters of rSAPV peptidase, SPVP, Thermolysin type X, and Alcalase 2.4 L FG enzymes were estimated by assaying the enzymatic activity using an increasing concentration of casein and Suc-F-A-A-F-*p*NA at pH 9 and 60°C, see Table 5. Nevertheless, the *K*_*m*_ and *V*_max_ values of rSAPV were estimated to be 0.313 mM and 74,652 U/mg, respectively. Lower *K*_*m*_ value indicates more affinity towards the substrate. With casein as substrate, the *k*_cat_*/K*_*m*_ of rSAPV was at least 2.6, 10.3, 7.9, and 9.8 folds higher, respectively, than those observed for SPVP, Thermolysin type X, Alcalase 2.4 L FG, and Bioprotease N100L, while with Suc-F-A-A-F-*p*NA as the substrate, the *k*_cat_*/K*_*m*_ of rSAPV was at least 1.9, 9.3, 6.9, and 9.2 folds higher, respectively, than those observed for SPVP, Thermolysin type X, Alcalase 2.4 L FG, and Bioprotease N100L (Table 5).

#### 3.7.3. Effects of Organic Solvents on Peptidase Activity and Stability

**Figure 4:**
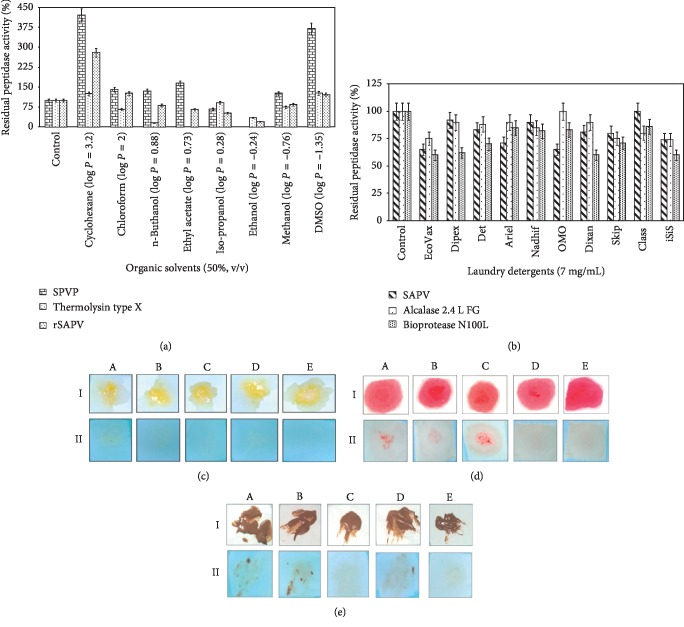
(a) Effect of organic solvents on the stability of the purified rSAPV, SPVP, and Thermolysin type X. Various organic solvents, with different log *P* values (50%, v/v), were tested at 190 strokes per min and 37°C for 3 days to evaluate their effects on peptidase stability. The residual peptidase activities were assayed under the same conditions of each enzyme. The activity of the enzyme without any organic solvent was taken as 100%. The activity is expressed as a percentage of activity level in the absence of organic solvents. Each point represents the mean of three independent experiments. Performance evaluation of the purified SAPV peptidase. (b) Stability of rSAPV, Alcalase 2.4 L FG, and Bioprotease N100L in the presence of liquid and solid laundry detergents. The list of liquid detergents included: Class (EJM, Sfax, Tunisia), EcoVax and Dipex (Klin Productions, Sfax, Tunisia), Skip (Unilever, France), and Nadhif (Henkel-Alki, Tunisia). The solid detergents used were OMO (Unilever, France), Det (Sodet, Sfax, Tunisia), Dixan (Henkel-Alki, Tunisia), iSiS (Henkel, Algiers, Algeria), and Ariel (Procter & Gamble, Switzerland). Peptidase activity of the control sample, which contained no additive and incubated under similar conditions, was taken as 100%. Vertical bars indicate standard error of the mean (*n* = 3). Washing performance analysis of rSAPV and commercial enzymes (500 U/mL) using egg (c), blood (d), and chocolate (e) stains with Class detergent (7 g/L). Stained cloths were washed with tap water (A), Class detergent (B), Class added with Alcalase 2.4 L FG (C), Class added with Bioprotease N100L (D), and Class added with rSAPV (E). I: untreated clothes (control) and II: treated clothes.

The effects of organic solvents on the activity and stability of the purified SPVP and Thermolysin type X proteases are shown in Figure 4(a). When compared with SPVP and Thermolysin type X peptidases, rSAPV enzyme is naturally stable and exhibits high peptidase activities in the presence of organic solvents. So, it would be very useful for organic synthetic reactions [17]. In upgrading environmentally mild chemical synthesis, the proteases are foreseen as supreme enzyme as the protease in nonaqueous media offers various novel characteristics as compared with traditional aqueous enzymatic transformation [49, 50]. The proteolytic enzymes have attracted a high deal of attention in organic synthesis because they have many advantages associated with the application of enzymes for the synthesis of peptides and esters [51–53]. Very few research studies are characterized in the literature concerning the screening of microorganisms which produce organic solvent-stable proteases [54]. Poor stability and low catalytic activity of enzymes are the limitations of their usage in organic solvents, which tend to strip water from protein and thereby disrupt noncovalent forces and decrease enzyme activity and stability. The ability to use enzymes in nonaqueous solvents expands the potential applications of biocatalysts in chemical transformations, which is useful for many industries. Enzymatic reactions in organic solvents provide numerous industrially attractive advantages, such as increased solubility of nonpolar substrates, reversal of the thermodynamic equilibrium of hydrolysis reactions, suppression of water-dependent side reactions, alternation of substrate specificity and enantioselectivity, and elimination of microbial contamination.

#### 3.7.4. Stability and Compatibility of Peptidase with Laundry Detergents

As illustrated in Figure 4(b), rSAPV peptidase is compatible with all the tested commercial detergents at concentration of 7 g/L. It exhibited better stability with Class followed by Dipex, Nadhif, Det, Dixan, Skip, iSiS, Ariel, EcoVax, and OMO. rSAPV retained 100% of its initial activity with Class (vs 86% for Bioprotease N100L and 80% for Alcalase 2.4 L FG) even after 1 h incubation at 40°C. The SPVP protease retained about 60–100% of its initial activity in various commercial detergents [17]. The protease from *Virgibacillus pantothenticus* showed excellent compatibility in presence of the locally available detergents. It was noted to be stable in the presence of UltraVim, Ghari, and Wheel after 3 h of incubation at 40°C [33]. In addition, the protease from strain VIT P14 is stable in the presence of some detergents (Henko, Ariel, Rin, Tide, Surfexcel, and Technobright) [34].

Thus, the present study aimed at exploiting the application of a peptidase from moderately halophilic strain which revealed compatibility and stability under various liquid and solid detergents tested and therefore has a great potential in many laundry formulations.

#### 3.7.5. Removal of Blood Stains from Cotton Fabrics

Protein stains like chocolate, egg, and blood have been hard to eliminate with commercial detergent. Some stains could only be dealt with at high temperatures and even then the stain was only partly removed. Detergent enzymes especially with proteases offered a key and are being persistently improved to digest proteinaceous stains. The ability of proteases to hydrolyse the various proteinaceous stains has attracted the interest of industrialists in detergent market. As shown in Figures 4(c)–4(e), the visual comparison of the washed cloth revealed that the combinations of every enzyme individually with commercial detergents (Class) yielded fairly good results of its ability to remove blood, egg, and chocolate stains. In fact, a limited washing performance was observed with tap water only or with detergent (Class) only. The supplementation of SAPV enzyme or commercial proteases (Bioprotease N100L or Alcalase 2.4 L FG) in Class detergent seems to enhance the cleaning process as evidenced by rapid stain removal. Thus, SAPV peptidase was characterized by its strong hydrolytic effect against blood and chocolate which are recalcitrant stains. Similarly, protease from strain VIT P14 demonstrated its potential use as a bioadditive detergent in the removing of the blood stains from cotton cloth [34]. Although reported the usefulness of thermostable alkaline peptidase from *Bacillus* and related genus for the removal of protein stains from cotton cloth in the presence and absence of detergents [17, 24, 31], we believe that the SAPV peptidase form is more effective.

## 4. Conclusions

In this research, a SAPV peptidase was produced by utilizing shrimp shell by-product as the energy and carbon sources, and the peptidase was successfully purified to homogeneity by two steps. Biochemical properties of the purified SAPV enzyme demonstrated that it has a high optimum working temperature and pH, which could make the peptidase suitable for industries applications. This was supported by cloning amino acid sequence inspection and homology modeling of the gene encoding SAPV peptidase, which is endowed with a number of characteristics that are highly valued for the detergent industry and peptide synthesis. These observations inspired us to explore other enzymes from strain FarD^T^. The structure-function relationship of SAPV is now underway to improve the properties of this peptidase. Further works will be investigated in our laboratories to perform the crystallization of SAPV enzyme.

## Figures and Tables

**Table 1:** Flow sheet purification of the SAPV enzyme from *Virgibacillus natechei* strain FarD^T^.

Purification step^a^	Total activity (units)^b^ ×10^3^	Total protein (mg)^b,c^	Specific activity (U/mg of protein)^b^	Purification yields (%)	Purification factor (fold)
Crude extract	8,000 ± 120	3,624 ± 71.3	2,207	100	1
(NH_4_)_2_SO_4_ fractionation (20–80%)-dialysis	6,640 ± 54	1,382 ± 41.2	4,804	83	2.17
HPLC (ZORBAX PSM 300 HPSEC)	4,864 ± 22	65.15 ± 0.83	74,652	60.8	33.82

^a^Experiments were conducted three times, and ± standard errors are reported. ^b^One unit of peptidase activity was defined as the amount of enzyme required to release 1 *µ*g tyrosine per minute under the experimental conditions used. ^c^Amounts of protein were estimated by the method of Bradford [27].

**Table 2:** Alignment of the NH_2_-terminal amino acid sequence of the peptidase SAPV from *Virgibacillus natechei* strain FarD^T^ with the NH_2_-terminal amino acid sequences of other *Bacillus* peptidases.

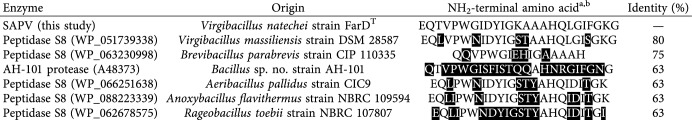

^a^Amino acid sequences for comparison were obtained using the program BLASTP (NCBI, NIH, USA) database. The GenBank accession number is in parentheses. ^b^Residues not identical with the SAPV peptidase from *Virgibacillus natechei* strain FarD^T^ are shaded.

**Table 3:** Effects of synthetic and natural inhibitors, with a molar ratio of [inhibitor]/[enzyme] was 100, as well as group-specific reagents and chelating agents, on SAPV stability. SAPV activity assayed without inhibitor or reducing agent was taken as control (100%). The nontreated and dialyzed SAPV was considered as 100% for metal ion assay. Residual SAPV activity was measured at pH 9 and 60°C.

Compound	Concentration	Residual peptidase activity (%)^a^
None	—	100 ± 2.5
PMSF	5 mM	0 ± 0.0
DFP	2 mM	0 ± 0.0
SBTI	3 mg/mL	96 ± 1.9
TLCK	1 mM	104 ± 2.6
TPCK	1 mM	108 ± 2.8
Benzamidine	5 mM	102 ± 2.6
DTNB	10 mM	117 ± 3.0
LD-DTT	1 mM	109 ± 2.6
2-ME	5 mM	113 ± 2.8
NEM	2 mM	100 ± 2.5
Iodoacetamide	5 mM	100 ± 2.5
Leupeptin	2 mM	111 ± 2.8
Pepstatin A	1 mM	102 ± 2.7
MIA	50 *µ*M	97 ± 2.1
EPNP	5 mM	104 ± 2.6
1,10-Phenanthroline monohydrate	10 mM	105 ± 2.7
EDTA	10 mM	86 ± 1.3
EGTA	2 mM	74 ± 1.2
Ca^2+^ (CaCl_2_)	2 mM	290 ± 7
Fe^2+^ (FeCl_2_)	2 mM	210 ± 4.8
Cu^2+^ (CuCl_2_)	2 mM	125 ± 2.8
Mn^2+^ (MnCl_2_)	2 mM	100 ± 2.5
Mg^2+^ (MgCl_2_)	2 mM	100 ± 2.5
Zn^2+^ (ZnCl_2_)	2 mM	120 ± 2.9
Co^2+^ (CoCl_2_), Hg^2+^ (HgCl_2_), or Cd^2+^ (CdCl_2_)	2 mM	0 ± 0.0

^a^Values represent the means of three replicates, and ± standard errors are reported. SBTI: soybean trypsin inhibitor; TLCK: *Nα*-*p*-tosyl-l-lysine chloromethyl ketone; TPCK: *Nα*-*p*-tosyl-l-phenylalanine chloromethyl ketone; DTNB: 5,5′-dithio-bis-2-nitro benzoic acid; NEM: *N*-ethylmalemide; MIA: monoiodoacetic acid; EPNP: 1,2-epoxy-3-(*p*-nitrophenoloxy) propane.

**Table 4:** Substrate specificity profile of rSAPV enzyme.

Substrate	Concentration	Absorbance (nm)^a^	Relative protease activity (%)^b^
*Natural protein*			
Casein	25 g/L	600	100 ± 2.5
Albumin	25 g/L	600	80 ± 1.8
Gelatin	25 g/L	600	42 ± 1.2
Ovalbumin	25 g/L	600	16 ± 0.4
Keratin	25 g/L	600	24 ± 0.9

*Modified protein*			
Azo-casein	20 g/L	440	100 ± 2.5
Albumin azure	20 g/L	440	64 ± 1.6
Keratin azure	20 g/L	440	18 ± 1.4
Collagen type I or II	10 g/L	440	0 ± 0.0

*Ester*			
ATEE	10 mM	253	100 ± 2.5
BTEE	10 mM	253	89 ± 2.4
BAEE	10 mM	253	0 ± 0.0
BCEE	10 mM	253	0 ± 0.0
TAME	10 mM	253	0 ± 0.0

*Synthetic peptide (pNA)*			
*P4-P3-P2-P1-P′1*			
Suc-F-*p*NA	5 mM	410	52 ± 1.7
Benz-Y-*p*NA	5 mM	410	0 ± 0.0
Met-*p*NA	5 mM	410	0 ± 0.0
Ac-L-*p*NA	5 mM	410	0 ± 0.0
Pro-*p*NA	5 mM	410	0 ± 0.0
Ac-A-*p*NA	5 mM	410	0 ± 0.0
Benz-R-*p*NA	5 mM	410	63 ± 1.9
Suc-Y-L-V-*p*NA	5 mM	410	0 ± 0.0
Suc-A-A-A-*p*NA	5 mM	410	0 ± 0.0
Suc-A-A-V-*p*NA	5 mM	410	21 ± 0.1
Suc-A-A-F-*p*NA	5 mM	410	35 ± 0.8
Benz-F-V-R-*p*NA	5 mM	410	0 ± 0.0
Suc-F-A-A-F-*p*NA	5 mM	410	100 ± 2.5
Suc-A-A-P-F-*p*NA	5 mM	410	87 ± 2
Suc-A-A-V-A-*p*NA	5 mM	410	75 ± 1.8
Suc-A-A-P-M-*p*NA	5 mM	410	62 ± 1.4
Suc-A-A-P-L-*p*NA	5 mM	410	38 ± 0.4
Suc-L-L-V-Y-*p*NA	5 mM	410	52 ± 0.9
Ac-Y-V-A-D-*p*NA	5 mM	410	0 ± 0.0

^a^Values represent the means of three replicates, and ± standard errors are reported. ^b^The activity of each substrate was determined by measuring absorbance at specified wave lengths according to the relative method reported elsewhere [32]. BTEE: *N*-benzol-l-tyrosine ethyl ester; ATEE: *N*-acetyl-L-tyrosine ethyl ester monohydrate; BAEE: *N*-benzol-L-arginine ethyl ester; BCEE: *S*-benzyl-L-cysteine ethyl ester hydrochloride; TAME: *Nα*-*p*-tosyl-L-arginine methyl ester hydrochloride. *N*-succinyl-L-Phe-*p*-nitroanilide; *N*-benzoyl-L-Tyr-*p*-nitroanilide; *N*-acetyl-L-Leu-*p*-nitroanilide; L-Met-*p*-nitroanilide; L-Pro-*p*-nitroanilide trifluoroacetate salt; *N*-acetyl-L-Ala-*p*-nitroanilide; L-Val-*p*-nitroanilide hydrochloride; *N*-benzoyl-L-Arg-*p*-nitroanilide; *N*-succinyl-L-Tyr-L-Leu-L-Val-*p*-nitroanilide; *N*-succinyl-L-Ala-L-Ala-L-Ala-*p*-nitroanilide; *N*-succinyl-L-Ala-L-Ala-L-Phe-*p*-nitroanilide; *N*-succinyl-L-Ala-L-Ala-L-Val-*p*-nitroanilide; *N*- benzoyl-L-Phe-L-Val-L-Arg-*p*-nitroanilide; *N*-succinyl-L-Phe-L-Ala-L-Ala-L-Phe-*p*-nitroanilide; *N*-succinyl-L-Ala-L-Ala-L-Pro-L-Phe-*p*-nitroanilide; *N*-succinyl-L-Ala-L-Ala-L-Val-L-Ala-*p*-nitroanilide; *N*-succinyl-L-Leu-L-Leu-L-Val-L-Tyr-*p*-nitroanilide; *N*-succinyl-L-Ala-L-Ala-L-Pro-L-Met-*p*-nitroanilide; *N*-succinyl-L-Ala-L-Ala-L-Pro-L-Leu-*p*-nitroanilide; *N*-acetyl-L-Tyr-L-Val-L-Ala-L-Asp-*p*-nitroanilide.

**Table 5:** Kinetic parameters of purified peptidases: rSAPV, SPVP, Thermolysin type X, Alcalase 2.4 L FG, and Bioprotease N100L for hydrolysis of natural protein (casein) and synthetic peptide (Suc-F-A-P-F-*p*NA).

Substrate	Enzyme	*K*_*m*_ (mM)^a^	*V*_max_ (U/mg)^a^	*k*_cat_ (min^−1^)	*k*_cat_/*K*_*m*_ (min^−1^ mM^−1^)
Casein	rSAPV	0.313 ± 0.01	74,652 ± 570	49,768	159,003
	SPVP	0.455 ± 0.03	41,255 ± 425	27,503	60,446
	Thermolysin type X	0.738 ± 0.05	17.126 ± 150	11,417	15,470
	Alcalase 2.4 L FG	0.765 ± 0.06	22,915 ± 285	15,277	19,970
	Bioprotease N100L	0.800 ± 0.07	19,500 ± 113	13,000	16,250

Suc-F-A-A-F-*p*NA	rSAPV	0.600 ± 0.04	155,400 ± 990	103,600	172,667
	SPVP	0.700 ± 0.05	95,833 ± 596	63,889	91,270
	Thermolysin type X	1.100 ± 0.09	30,700 ± 392	20,467	18,606
	Alcalase 2.4 L FG	1.410 ± 0.15	52,950 ± 495	35,300	25,035
	Bioprotease N100L	1.560 ± 0.21	43,900 ± 490	29,267	18,761

^a^Values represent the means of three replicates, and ± standard errors are reported.

## Data Availability

The datasets generated and/or analyzed during the current study are available on the GenBank repository, https://www.ncbi.nlm.nih.gov/genbank/. The GenBank accession number for the nucleotide sequence of the *sapV* gene referred to in the text is **MN094794**. Other datasets generated during and/or analyzed during the current study are available from the corresponding author on reasonable request.
